# A New Approach in Meat Bio-Preservation through the Incorporation of a Heteropolysaccharide Isolated from *Lobularia maritima* L.

**DOI:** 10.3390/foods11233935

**Published:** 2022-12-06

**Authors:** Boutheina Ben Akacha, Basma Najar, Francesca Venturi, Mike Frank Quartacci, Rania Ben Saad, Faiçal Brini, Wissem Mnif, Miroslava Kačániová, Anis Ben Hsouna

**Affiliations:** 1 Laboratory of Biotechnology and Plant Improvement, Centre of Biotechnology of Sfax, University of Sfax, Sfax 3018, Tunisia; 2 Department of Agriculture, Food and Environment, University of Pisa, Via del Borghetto 80, 54126 Pisa, Italy; 3 Department of Chemistry, College of Sciences and Arts in Balgarn, University of Bisha, P.O. Box 199, Bisha 61922, Saudi Arabia; 4 Institute of Horticulture, Faculty of Horticulture, Slovak University of Agriculture, Tr. A. Hlinku 2, 949 76 Nitra, Slovakia; 5 Department of Bioenergy, Food Technology and Microbiology, Institute of Food Technology and Nutrition, University of Rzeszow, 4 Zelwerowicza St, 35601 Rzeszow, Poland; 6 Department of Environmental Sciences and Nutrition, Higher Institute of Applied Sciences and Technology of Mahdia, University of Monastir-Tunisia, Monastir 5000, Tunisia

**Keywords:** polysaccharides, *Lobularia maritima* L., bioactive compounds, bio-preservation, foodborne pathogens

## Abstract

In this study, a new heteropolysaccharide extracted from *Lobularia maritima* (L.) Desv. (*Lm*PS), a halophyte harvested in Tunisia, was evaluated as an antioxidant and antibacterial additive in the bio-preservation of raw minced meat. For antibacterial testing, Gram-positive bacteria such as *Staphylococcus aureus* ATCC and *Listeria monocytogenes* ATCC 19,117 and Gram-negative bacteria such as *Salmonella enterica* ATCC 43,972 and *Escherichia coli* ATCC 25,922 were used. The results indicate that this polymer had a significant antibacterial activity against foodborne pathogens. Additionally, the effects of *Lm*PS at 0.15, 0.3 and 0.6% on refrigerated raw ground beef were investigated from a microbiological, chemical, and sensory perspective. Microbiological analysis of the meat showed that treatment with *Lm*PS significantly (*p* < 0.05) improved its shelf life, while the biochemical analysis evidenced a significant (*p* < 0.05) decrease in lipid oxidation. *Lm*PS at 0.6% significantly reduced by 61% and 48% metmyoglobin accumulation at the end of the storage period when compared to BHT and control samples, respectively. The chemometric approach highlighted the relationships among the different meat quality parameters. *Lm*PS can be introduced in the food industry as a powerful natural additive and could be an alternative to synthetic antioxidant compounds.

## 1. Introduction

A growing number of processing approaches are being developed by the food industry to meet consumer’s demands. Food safety and cleanliness are persistent concerns for the meat processing industry. Food degradation during refrigeration is mainly caused by microbiological growth and oxidative rancidity [1,2]. Increasing awareness of the importance of healthy and safe nutrition has forced the food industry to correctly label their products and to encourage consumers to avoid synthetic additives such as BHA (butylated hydroxyanisole), BHT (butylated hydroxytoluene) and tertiary butylhydroquinone (TBHQ) (E-number 319, food additive). These additives are generally used to prevent food spoilage, although their use is restricted in different countries or organizations (the United States of America, the European Union (EU), etc.) due to their possible cytotoxic and carcinogenic effects [3,4]. This prompts the food industry to search for natural antioxidant preservatives [5,6]. Plants may be a main source of natural antioxidants since nearly all plants contain antioxidants that play a role in protecting against solar radiation and pests, as well as regulating chemical energy production [7]. Over the past few years in the food industry there has been a growing interest in polysaccharides from plants, animals and microbes [8,9]. Polysaccharides derived from plants may be effective antioxidants because they scavenge initial radicals, interrupt chain reactions, decompose peroxides, and contrast free radicals. In addition, they are natural substances with little or no adverse effects [10].

The non-toxic and biocompatible properties of plant-derived polysaccharides have been exploited by researchers as food hydrocolloids with potential rheological and antioxidant effects to prevent and control oxidation processes in meat products [11]. Furthermore, these bioactive molecules improve the nutritional benefits of many foods based on their concentration [12]. 

Glucose, galactose, and xylose were the main components of *Lm*PS according to our previous study [13]. The in vivo tests evidenced a protective effect of *Lm*PS against CCL4-induced hepatotoxicity in rats. This protective potential of *Lm*PS could be linked to a high antioxidant capacity. Indeed, this polymer showed a significantly high antioxidant activity, with an EC_50_ value of 0.2 mg/mL which was lower than that of catechin (0.25 mg/mL), used as a standard for DPPH scavenging activities.

This work aims to verify the effectiveness of *Lm*PS as a preservative for refrigerated ground beef meat in comparison with BHT. The microbiological and oxidative stability of the treated meat were evaluated as well as the sensory profile, while raw meat was used as a control. The results were analysed by multivariate statistical analysis to evaluate the relationships among oxidative stability, microbiological measurements, and sensory properties.

## 2. Materials and Methods

### 2.1. Polysaccharide Availability

*Lm*PS was extracted from the aerial parts of *Lobularia maritima* (collected in March 2020 in the Chebba area (Mahdia, Tunisia, latitude 35.23°, longitude 11.11°). At the end of the extraction process, the resulting material contained a high amount of carbohydrates (~85%) and the calculated medium molecular weight was 130.62 kDa. Acute toxicity tests on *Lm*PS-treated rats showed no toxicity, with a LD_50_ above 250 mg/kg [13]. 

### 2.2. Bacterial Strains Origin and Cell Culture

Two Gram-positive (G^+^) [*Staphylococcus aureus* ATCC 25,923 and *Listeria monocytogenes* ATCC 19,117] and two Gram-negative (G^−^) [*Salmonella enterica* ATCC 43,972 and *Escherichia coli* ATCC 25,922] bacteria stains were used to evaluate the antibacterial efficacy of *Lm*PS. The bacterial strains origin as well as cell culture were previously reported by Ben Hsouna [5]. Briefly, bacteria were obtained from both the international collections of the American Type of Culture Collection (ATCC) and the local collection of cultures from the Center of Biotechnologie of Sfax (Tunisia). The bacterial progeny was grown on MH agar (Bio-Rad, Marnes-la-Coquette, France) at a temperature of 37 °C for 12–14 h. The inoculum preparation was carried out using an overnight broth culture by dilution in saline solution to 10^6^ colony-forming units (CFU/mL).

### 2.3. Determination of Antibacterial Activity

Antibacterial tests were performed as described by Ben Hsouna et al. [5] and the broth microdilution test by the sterile Mueller–Hinton media (Bio-Rad, Marnes-la-Coquette, France). One hundred μL was evenly spread on the surface of MH agar plates (Oxoid Ltd., Basingstoke, UK). Wells were dug into the agar using a sterile Pasteur pipette. The final concentration was 50 mg/mL of *Lm*PS dissolved in distilled water. Then, 50 μL of extract was placed into the wells and the plates were incubated for 24 h at 37 °C. Streptomycin (20 μg/well) and distilled water were used as positive and negative controls, respectively. The antimicrobial effect was checked measuring the diameter of the circular inhibition zones of the wells. The tests were carried out in triplicate.

### 2.4. Determination of MIC and MBC

The determination of the minimum inhibitory concentrations (MICs) of *Lm*PS followed the method previously reported by Ben Hsouna et al. [5]. Briefly, the evaluation was based on the broth microdilution method (96 microplates) by adding 10 μL of cell suspension and 25 μL of thiazolyl tetrazolium blue bromide (0.5 mg/mL) (Sigma-Aldrich, Taufkirchen, Germany) to all wells followed by an incubation for 30 min. The marker of the growth of microorganisms, the thiazolyl tetrazolium blue bromide, is a salt that acts as an electron acceptor and, in the presence of biologically active organisms, is reduced to red-coloured formazan. MIC (%) was evaluated as the lowest concentration of *Lm*PS that inhibited the evident growth of each tested bacteria. 

Ten microliters were taken from each well and inoculated into strata MH plates to calculate the minimum bactericidal concentration of *Lm*PS (MBCs). The count of surviving organisms was carried out after 24 h of incubation at 37 °C. MBC was determined as the lowest concentration of *Lm*PS at which 99% of the bacteria were killed. MICs and MBCs experiments were performed in triplicate.

### 2.5. Meat Samples Preparation and Conditioning

Fresh beef samples were obtained from a regional slaughterhouse in Sfax (Tunisia). The samples were then minced using a sterile meat grinder and split into five lots as shown in the Table 1 below: 

### 2.6. Microbial Count Determination

Microbiological evaluation was carried out according to ISO 7218 [14] after 14 days of storage at 4 °C. In short, a homogenization of 25 g of samples in 225 mL of sterile NaCl solution (0.85%) was carried out for 10 min. For microbial counts, decimal dilutions of the samples to be spread on the corresponding medium were prepared. Aerobic plate counts (APC) were performed by plate count agar (PCA, Oxoid, Basingstoke, UK) and incubated at 30 °C for 48 h [15]. Aerobic psychrotrophic counts (PTC) were calculated as described above for APC, except that plates were incubated at 7 °C for 10 days [16]. Enterobacteriaceae were enumerated on a violet red bile lactose agar(VRBL, Oxoid, Basingstoke, UK), and incubated at 37 °C for 24 h [17].

### 2.7. Physicochemical Analysis

#### 2.7.1. pH 

The pH value was determined using a pH meter (Model: YK-21PH) by inserting the electrode directly into 5 g of filtrate from a sample of raw ground meat that has been previously vortexed in distilled water (pH = 7) at each sampling stage [5]. 

#### 2.7.2. Lipid Oxidation

The extent of lipid oxidation in the different samples was evaluated by the determination of thiobarbituric acid reactive substances (TBARS) such as malondialdehyde (MDA). TBARS were assessed as reported by Eymard et al. [18]. The results were expressed in milligrams of malonaldehyde equivalents per kilogram of sample (mg/kg).

#### 2.7.3. Metmyoglobin (MetMb) Analysis

The metMb content was determined following the method described by Wang et al. [19]. Five g of sample were mixed with 25 mL of cold 0.04 M K_3_PO_4_ buffer (pH 6.8). Meat samples were homogenized and kept for 1 h in an ice bath, after which they were stored for 1 h at a refrigerated temperature (4 °C). Then, samples were centrifuged for 30 min at 4500 rpm in a refrigerated centrifuge (Eltek MP-400-R Eltek India, Delhi, India) at 4 °C. The supernatant was collected and filtered through Whatman filter paper No. 42. The absorbance was determined at 525 (A525), 572 (A572), and 700 (A700) nm. The metMb was expressed as a percentage and was calculated as follows:

MetMb% = [−2.51 (A572*/*A525) + 0.777 (A565*/*A525) + 0.8 (A545*/*A525) + 1.098] × 100

### 2.8. Sensory Profile

To determine the maximum storage time during which the organoleptic properties of the stored meat were maintained above their acceptability limit as a function of preserving method used, the method described by Hsouna et al. [5] was used. Specifically, ten panellists were recruited and trained among students and employees belonging to the University of Sfax and they were asked to evaluate the organoleptic quality of each meat sample based on colour, odour, appearance, and overall acceptability. For each sensory attribute (colour, odour and overall acceptability), a 9-point hedonic scale ranging from 9 (like much) to 1 (dislike much) was used, with 5 indicating the “acceptability limit”. As a function of the preserving method, the sensory shelf-life of the stored samples was determined when a single parameter fell below the acceptability limit. Each organoleptic test was arranged in the morning, in a relaxed atmosphere. Batches of 10 g raw ground beef were divided in white Styrofoam plates and presented to panellists with codes in a random order.

The selection and training of assessors were performed according to the Department of Agriculture, Food and Environment (DAFE) of the University of Pisa internal procedure as briefly described below:

1. Theoretical introduction to the principles of human physiology of sight, smell, and taste.
2. Arrangement of preliminary training tests, mainly based on the utilization of model standard solutions, to collect information about the tasting capacity of each assessor (i.e., sensory acuity, odour and flavour memory, term use and recall, scoring consistency).
3. To harmonize the assessment as well as to select the main descriptors to be used during experimental panel test, a preliminary consensus panel was carried out in the morning, in a well-ventilated quiet room and in a relaxed atmosphere to evaluate different meat samples at different storage times.

### 2.9. Statistical Analysis

A randomize block design of all treatments was used for the statistical analysis. A two-way analysis of variance (ANOVA) was conducted for all parameters, except for sensory analysis, using the SPSS 20 statistical package.

The Durbin–Watson statistical test (*p* < 0.05) was performed to assess the presence of autocorrelation between overall acceptability and sensory characteristics. Autoscaled data were used for the chemometric analysis. Principal compound analysis (PCA) and hierarchical cluster analysis (HCA) were performed to discriminate among samples. All analyses were carried out using the XLSTAT software for Windows (v.2022.1.08, Addinsoft, New York, NY, USA).

## 3. Results and Discussion

### 3.1. Anti-Foodborne-Pathogen Activity of LmPS

Even though their mechanisms of action are still not well understood [20], polysaccharides from plants and animals have been studied for their antibacterial capacity. In this context, the antibacterial activities of the novel heteropolysaccharide from *L. maritima* were evaluated against G^+^ (*S. aureus* ATCC 25,923 and *L. monocytogenes* ATCC 19,117) and G^−^ (*E. coli* ATCC 25,922 and *S. enterica* ATCC 43,972) bacteria. 

The results of the antibacterial efficacy of *Lm*PS (Table 2) highlight the inhibitory effects of *Lm*PS against the tested strains. *Lm*PS extracts showed a powerful antimicrobial activity against *E. coli*, *S. enterica*, and *L. monocytogenes*, with inhibition zones of 29.5 ± 0.3, 31.0 ± 0.3 and 31.5 ± 0.1 mm, respectively. The inhibitory zones for streptomycin (20 µg/well), used as a positive control, were 27.5, 26.2, and 27.0 mm on the same strains. Our results are higher than those obtained by Han et al. [21], who evaluated the antibacterial capacity of polysaccharides extracted from the fruits of *Broussonetia papyrifera*.

Bacterial cell walls are attacked by polysaccharides which act as a barrier, preventing nutrients from entering bacteria cells and inhibiting growth]. The barrier effect was reported to be concentration dependent [9,11]. These findings agree with those reported herein. Additionally, it has been suggested that disk diffusion results might be influenced by the polysaccharide diffusion capacity [8,22,23]. Thus, with a low molecular weight, *Lm*PS may have a higher diffusion capacity. 

The broth microdilution method was also used to assess the in vitro activity of *Lm*PS, and the results were expressed as minimum inhibitory concentration (MIC) and minimum bactericidal concentration (MBC). The data shown in Table 3 indicate that *Lm*PS displayed different levels of antimicrobial activity against the evaluated food pathogenic bacteria. Despite this, no significant difference (*p* < 0.05) was evident in the antibacterial activities, regardless of being G^−^ or G^+^ bacteria. MIC values ranged between 170–150 µg/mL and 120–150 µg/mL, respectively. According to these results, *L. maritima* polysaccharides exhibit an interesting new potential antibacterial in the food industry. According to the CMB/CMI ratio values, *Lm*PS presented a bacteriostatic effect against the four pathogenic strains. Therefore, *Lm*PS has the same mechanism of action as the antibacterial agents that act by inhibiting bacterial protein synthesis, and thus may have several medical applications. Because they only inhibit bacterial growth, bacteriostatic antimicrobials require a functioning host immune system to eliminate overgrowth [24].

Over the past decades a variety of plant polysaccharides has been taken into consideration for their important bioactivities. Some plant polysaccharides have a strong antibacterial activity against different G^+^ and G^−^ bacteria [22]. The antibacterial activity of plant polysaccharides can be exerted by enhancing cell membrane permeability, inhibiting the adsorption of pathogenic bacteria onto host cells, or by blocking the transmembrane transport of nutrients and energy [21]. Indeed, Wang et al. [25] reported that the antibacterial mechanisms of polysaccharides were shown to be via damaging cellular structural and inhibiting bioenergetics metabolism. Nevertheless, according to Zhou et al. [26] the antibacterial activity of plant polysaccharides can be exerted by increasing the permeability of the cell membrane, inhibiting the adsorption of pathogenic bacteria to host cells, or blocking the transmembrane transport of nutrients or energy substances. In general, six principle antibacterial mechanisms of polysaccharides have been investigated in previous studies, the most important of which are as follows: (i) effect on bacterial biofilm (e.g., chitosan [27,28]); exopolysaccharides extracts from *Pleurotus flabellatus* strain Mynuk mycelium [29]; sulphated polysaccharides extracted from *Chlamydomonas reinhardtii* [30]; xanthan-oligosaccharide [31]; probiotic bacteria exopolysaccharides [32]), (ii) effects on bacterial nucleic acids (e.g., FITC-labelled chitosan oligomers [33]; *Streptomyces virginia* H03 polysaccharide [22]; chitosan [34]; sulphated polysaccharides extracted from *Chlamydomonas reinhardtii* [30]), (iii) effects on bacterial intracellular metabolic pathways (e.g., Sulphated polysaccharides extracted from *Chlamydomonas reinhardtii* [30]; chitosan [28,35,36]; *Tetrastigma hemsleyanum Diels et Gilg’s* polysaccharide [35]), (iv) effects on bacterial mycoproteins (e.g., *Cordyceps cicadae* polysaccharide [36]; *Chaetomium globosum* CGMCC 6882 polysaccharide [37]; chitosan and chitosan oligosaccharides [38]; chitosan [34,39]), (v) effects on bacterial cell wall (e.g., chitosan [36], [40,41,42,43]; chitosan and chito-oligosaccharides mixture [43]; *Streptomyces virginia* H03 exopolysaccharide [22]; *Cordyceps cicadae* polysaccharide [36]), and (vi) effects on bacterial cell membrane (e.g., chitosan [39]; *Chaetomium globosum* CGMCC 6882 polysaccharide [44]; *Cordyceps cicadae* polysaccharide [36]; *Streptomyces virginia* H03 polysaccharide [22]; xanthan-oligosaccharide [31]; *Lactobacillus plantarum* and *Bacillus* spp. extracellular polysaccharides [45]).

### 3.2. LmPS Effect in Cold Storage Minced Beef Meat

Based on the finding of this work and others previously reported [12], *Lm*PS displayed interesting antimicrobial and antioxidant activities, hepatoprotective effects and antigenotoxic capacity. Therefore, it is believed that it is possible to apply *Lm*PS as a natural preservative in raw ground beef during refrigerated storage at 4 °C. To verify this, the effects of *Lm*PS on lipid and protein oxidations, microbial growth as well as sensory quality were investigated. For this, three concentrations of *Lm*PS were added to raw ground beef (0.15% (1*Lm*PS), 0.3% (2*Lm*PS) and 0.6% (3*Lm*PS)) which equals MIC, 2 × MIC, and 4 × MIC against *L. monocytogenes* ATCC 19,117, respectively.

#### 3.2.1. Microbiological Determination

Some foods are more correlated with foodborne illness and food poisoning than others. If contaminated, these foods can carry harmful germs which can seriously affect human health. Raw foods of animal origin, especially un- or undercooked meat and poultry, are the most susceptible to be infected [5]. The minimum contamination level of beef ground meat (APC) is about 2.1 ± 0.04 log_10_ CFU/g (Table 4). On the seventh day of cold storage at 4 °C, APC levels did not exceed 4 log_10_ CFU/g in the meat samples treated with *Lm*PS and BHT. The logarithmic evolution of APC in samples treated with different concentrations of *Lm*PS was suppressed and did not overcome the maximum recommended limit (6.7 log_10_ CFU/g) [6], even on day 14 of storage. Therefore, treatment with *Lm*PS at 0.6 % (*v/w*) protected minced meat from APC contamination, thus extending its shelf life. In contrast to the control and BHT samples, this limit was reached on days 10 and 14 of storage. Previous studies suggest that adding polysaccharides to meat and meat products as a natural preservative reduces APC scores and improves quality [13,14]. Our results agree with Kallel et al. [46] who pointed out the direct influence of polysaccharides from garlic straw extract on microbial growth in beef ground meat samples. These authors reported that these polysaccharides inhibited the growth of the total aerobic cell population for nine days. Thus, incorporating *Lm*PS into ground beef meat improves its microbiological quality during cold storage.

Temperature is a key factor for the microorganisms growth [47]. Storing foods at refrigeration temperature is becoming a regular practice to control the growth of psychrotrophic microorganisms, some pathogens, and to maintain the product worth. However, psychrotrophic germs are generally related to food spoilage at refrigeration temperatures [48]. In this regard, we monitored their level which, at the start of cold storage, was comparable in all samples and was about 2 log_10_ CFU/g.

The PTC evolution of the control samples increased significantly (*p* < 0.05) and reached higher values (Table 4) in comparison with the samples treated with *Lm*PS which at 0.3 and 0.6% (*v/w*) notably reduced PTC. Furthermore, at the end of the storage period 3*Lm*PS reduced PTC by 2.3 log_10_ CFU/g compared to the untreated (control) sample, PTC prolonging the shelf life to 14 days during the chilled storage. Therefore, 3*Lm*PS was able to better delay the growth of psychrotrophic bacteria compared to BHT and control samples.

In many food analyses, the number of *Enterobacteriaceae* can be used as an indicator of the sanitary status of food products [49]. As reported in Table 3, there was a variation in the *Enterobacteriaceae* levels of the samples. At the beginning the *Enterobacteriaceae* level of all samples was less than one. Throughout the storage period the raw minced beef meat containing 3*Lm*PS showed the lowest level (*p* < 0.05) compared to the control, BHT (0.01%), and the other two concentrations of *Lm*PS. The impact of *Lm*PS was related to dosage and storage duration. Ben Akacha et al. [6] reported that the treatment of minced beef meat with the essential oil of *L. maritima* at a rate of 0.076% resulted in high antimicrobial activities against APC, PTC and *Enterobacteriaceae*.

The results suggest that the addition of *L. maritima* heteropolysaccharides to raw minced beef meat at 0.6% (*v/w*) improves the shelf life of the product in comparison with the standard preparation including BHT as food preservative.

#### 3.2.2. Physicochemical Analyses

pH

**Figure 1:**
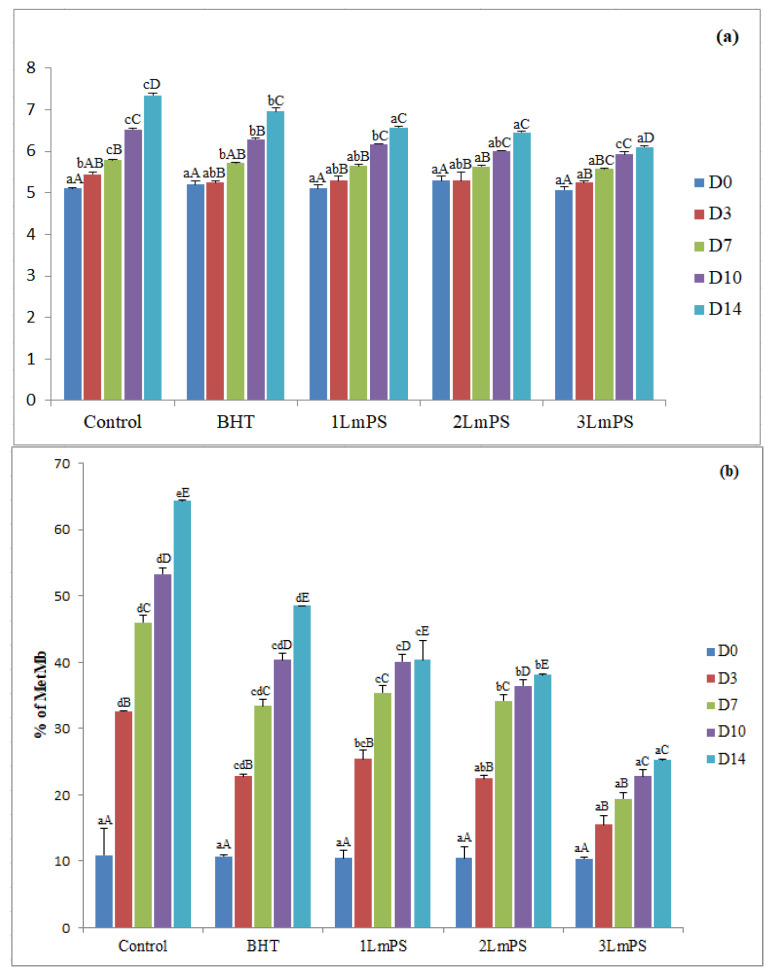

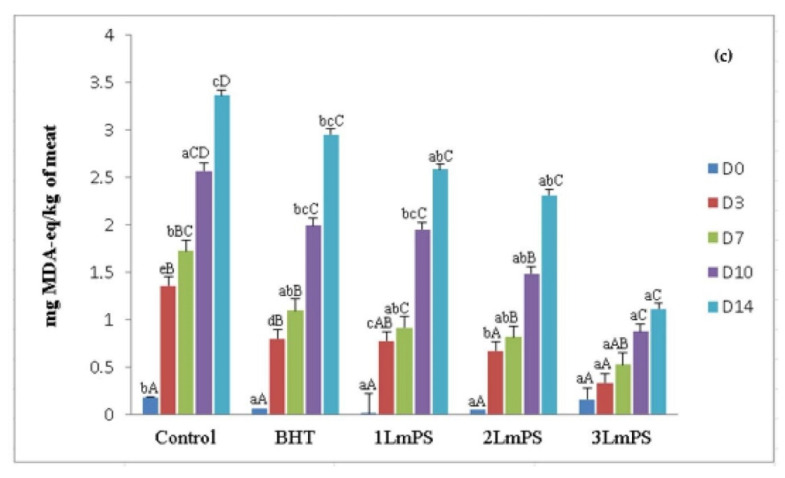
*Lm*PS incorporation effect on (**a**) pH, (**b**) metmyoglobin (%), and (**c**) thiobarbituric-acid reactive substances (TBARS) (mg malondialdehyde/kg) in crude minced meet within a shelf life of 14 days of storage at 4 °C. Mean ± standard deviation (*n* = 3). Letters a–d: values of the same storage day are significantly different (*p* < 0.05); letters A–D: values of the same concentration are significantly different.

The pH values of raw ground beef after 14 days of storage at 4 °C are presented in Figure 1a. The results show no significant difference (*p* > 0.05) for the different formulations at the beginning of the refrigeration period. During storage, the pH values increased substantially (*p* < 0.05) after inoculation of *Lm*PS into the ground beef. The pH values in the treated beef were meaningfully lower compared to the other treated samples (control and BHT) (*p* < 0.05); in particular, the 3*Lm*PS treatment showed a decrease compared to the control sample (*p* < 0.05). Alkaline compounds, protein degradation of bacterial metabolites and bacterial growth—mainly lactic acid contamination (*Clostridium*, *Bacillus*, *Lactobacillus* and *Enterococci*) in the muscle—caused pH increase [50].

Protein Oxidation

A water-soluble protein, myoglobin, affects the colour of meat. Meat discolouration and metmyoglobin formation are due to the depletion of the redox stability of the heme group through the loss of an electron during the oxidation processes [51]. The results show that at the beginning, the percentages of metMb were similar (higher than 10.4%) and increased over 14 days, which may be due to the denaturation of myoglobin [19] (Figure 1b). Lipids and myoglobin oxidation may produce by-products, such as peroxyl radicals and oxidized iron, which speed up the oxidation of proteins [52]. At the end of the storage, 3*Lm*PS showed the lowest metMb oxidation (25.3%), compared to the 1*Lm*PS, 2*Lm*PS, control, and BHT treatments (40.3%, 38.1%, 64.3%, and 48.6%, respectively). The loss of redness of the raw beef mince due to oxidation of oxymyoglobin to metMb [53] was delayed by the treatment with 3*Lm*PS which maintained the redness. These findings could be linked to the reduction of lipid oxidation, which in turn accelerated myoglobin oxidation [19].

Lipid Oxidation

Lipid oxidation is one of the primary non-microbiological causes of quality degradation in the production, storage, and distribution of foods, particularly meat and meat products. Off-aromas in meat mainly are related to TBARS. These substances represent secondary lipid oxidation products, mainly aldehyde and carbonyl derivatives of hydrocarbons [17,31,33]. The results evidence that TBARS levels in the different samples, independent of the treatment used, increased significantly (*p* < 0.05) during storage, though varying among the different treatments. On the first day of storage, no important changes (*p* > 0.05) in the TBARS levels of all raw beef ground meat samples were noted. TBARS values were higher in the control than in the treated samples during storage (Figure 1c). The lowermost TBARS values (1.11 mg malondialdehyde/kg) were reached by the 3*Lm*PS treatment after 14 days in comparison with the control and BHT (3.36 ± 0.07 and 2.95 ± 0.03 mg malondialdehyde/kg, respectively). It was reported that an index of two represents the cut-off point for the acceptability of oxidized beef [5]. The slowness of the lipid oxidation processes may be related to the increased antioxidant capacity of *Lm*PS, which had a significant inhibitory capacity against C18:2 peroxidation with a value close to that of catechin at 100 µg/mL [12]. Thus, the incorporation of 3*Lm*PS (0.6%) maintained the lowest TBARS values during the refrigerated preservation process at 4 °C. 

### 3.3. Sensory Profile 

**Figure 2:**
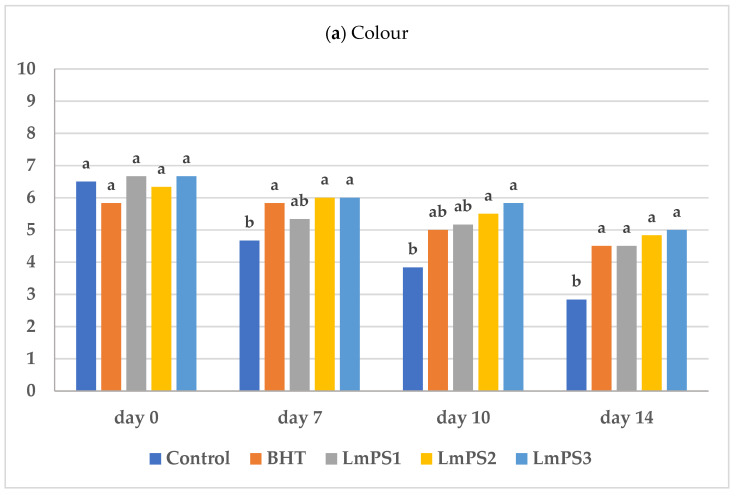

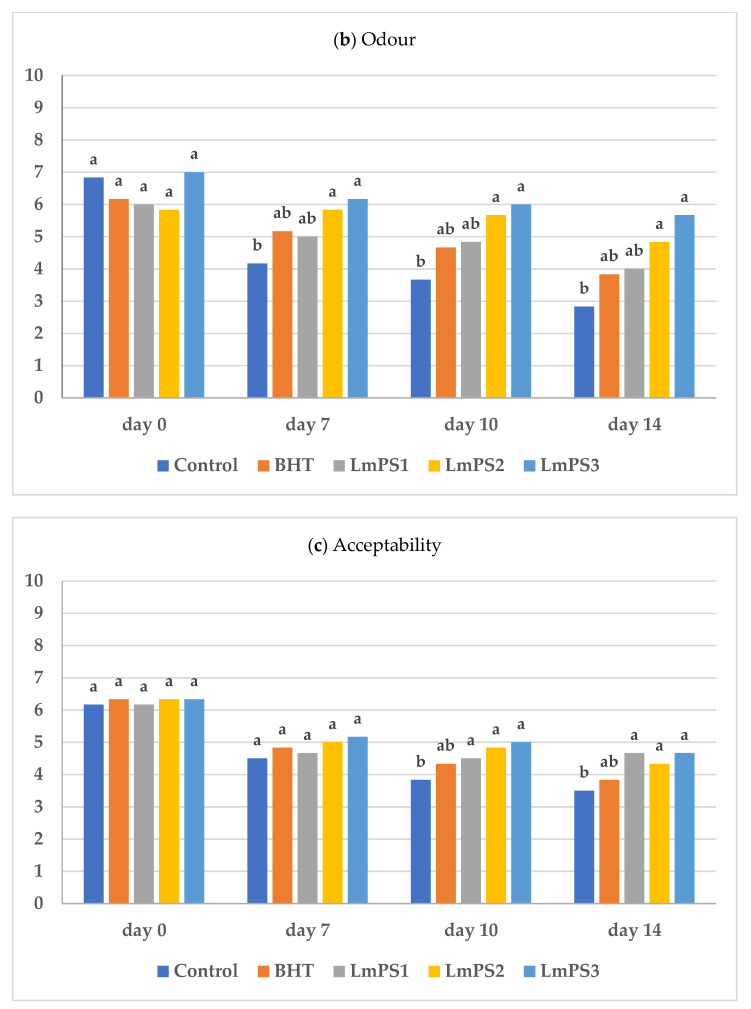
Organoleptic quality ((**a**) colour, (**b**) odour and (**c**) overall acceptability) of raw minced meat beef stored at T = 4 °C, using different preserving agents. In each group (same day of storage) different letters indicate mean values significantly different (*p* < 0.05).

As showed in Figure 2, without any preserving agent the organoleptic quality of meat was reduced significantly (*p* < 0.05) during storage because of the enzymatic browning between lipids and amino acids [31] that influenced negatively both colour and odour. After one week of storage, the control sample had all the evaluated parameters below the acceptability limit.

Interestingly, the lowest concentration of *Lm*PS showed the same preserving effect obtained with the BHT formulation. Furthermore, the higher the concentration of *Lm*PS added to the meat, the lower the quality decay rate during storage, suggesting that this polymer may have inhibited microorganism growth and lipid oxidation during the 14 days of refrigerated storage. 

The overall acceptability of the different samples decreased during storage, with all the samples below the acceptability limit at the end of the observation period. The sensory shelf life of the meat appeared extended when *Lm*PS was supplemented at 0.3% or more. In conclusion, due to the antioxidant and antibacterial activities of *Lm*PS, its incorporation at 0.6% in the raw ground beef could improve its organoleptic and nutritional properties.

### 3.4. Chemometric Approaches Underlying Lipid/Protein Oxidation and Microbial Growth

#### 3.4.1. Principal Component Analysis (PCA)

**Figure 3:**
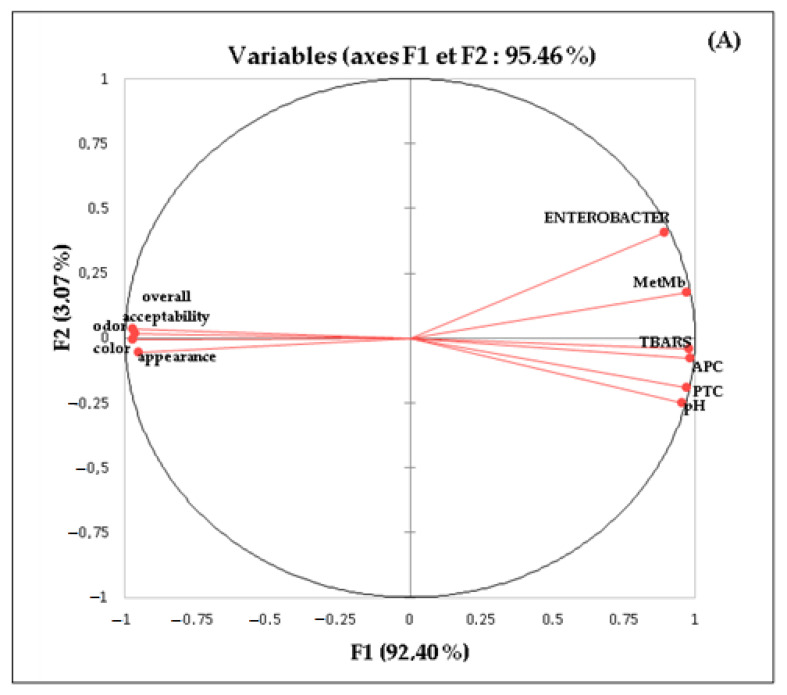

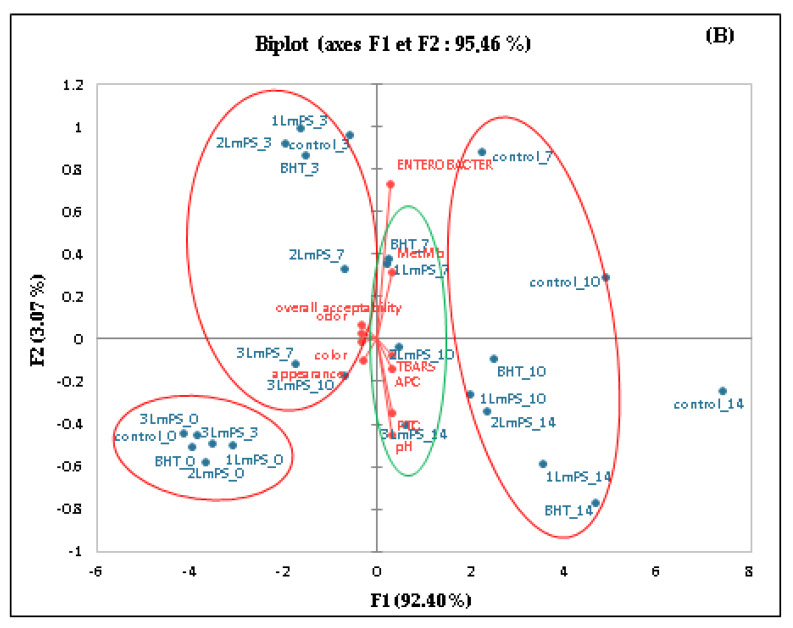
Principal component analysis (PCA) of meat data. (**A**) Scatterplot of PCA. (**B**) Biplot of PCA.

The data concerning the microbial, chemical, and sensory properties of the samples were analysed by PCA. The first component (F1 92.4%) explained most of the information (Figure 3). The (F1) axis (Figure 3A) was significantly correlated to most of the variables (metMb, TBARS, APC, overall acceptability, colour, appearance, and odour). On the contrary, the (F2) axis was more related to *Enterobacteriaceae* number, pH, and PTC. TBARS were strongly correlated with metMb (Figure 3A). It is reported that peroxyl radicals and oxidized iron, by-products of lipid and myoglobin oxidation, accelerate protein oxidation [54]. In addition, a positive relationship was observed between microbial growth and lipid/protein oxidation measurements. The findings herein are consistent with the effect of protein oxidation on sensory parameters. Based on Figure 3A, it can be suggested that oxidative stability as well as microbial loads played an important role in influencing the samples during storage. The graph concerning the factor scores (Figure 3B) points out a large difference among the 25 samples. Overall, the samples 3*Lm*PS*_14*, 2*Lm*PS*_10*, 1*Lm*PS*_7* and BHT_7 had low levels of protein oxidation (metMb), lipid oxidation (TBARS) and microbial growth. These samples were the most acceptable for the panellists. In contrast, the control was associated with high levels of lipid oxidation, protein oxidation and microbial growth. It is noteworthy that 3*Lm*PS was more efficient against microbial growth, delaying chemical oxidation, and thus extending the shelf life of ground beef.

#### 3.4.2. Two-Way Hierarchical Cluster Analysis

**Figure 4:**
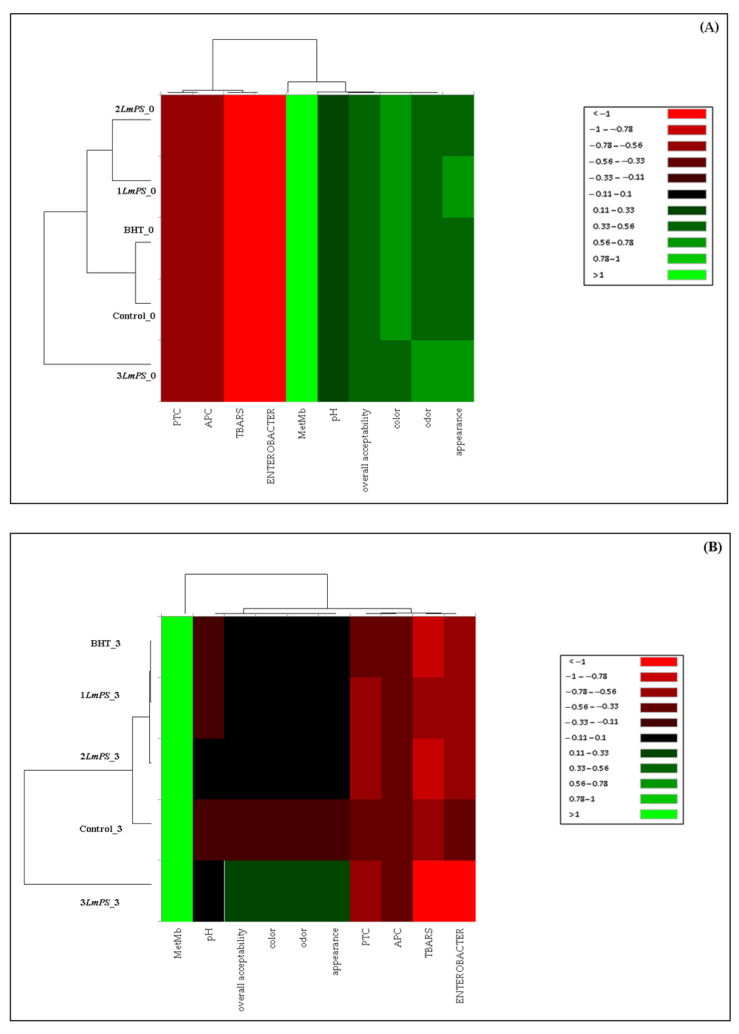

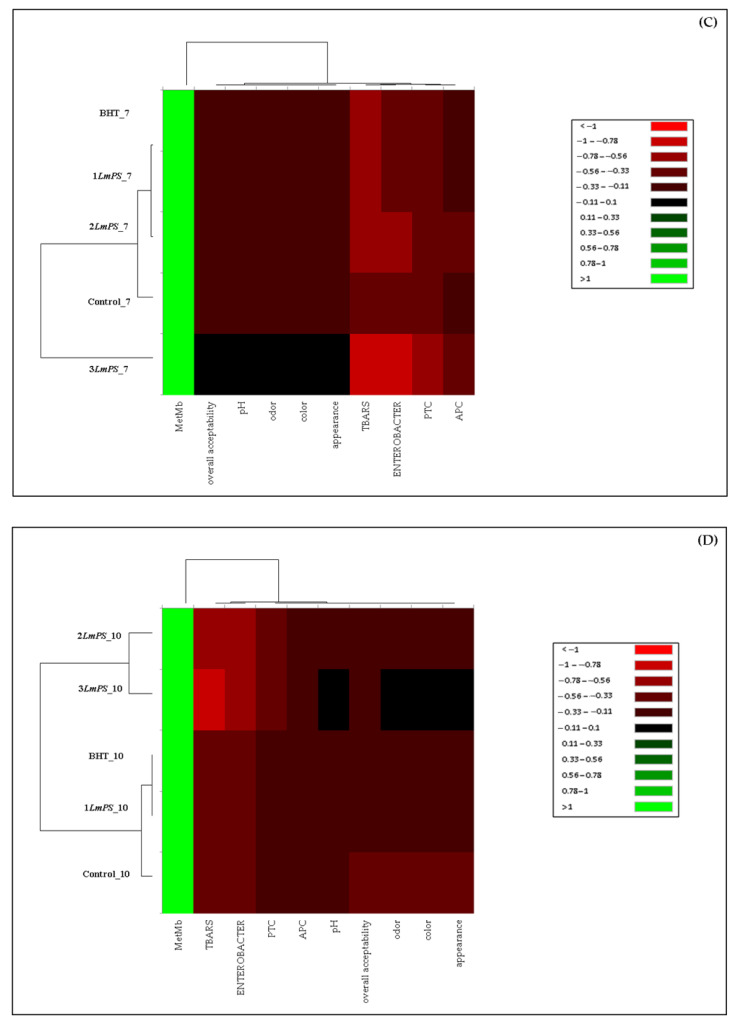

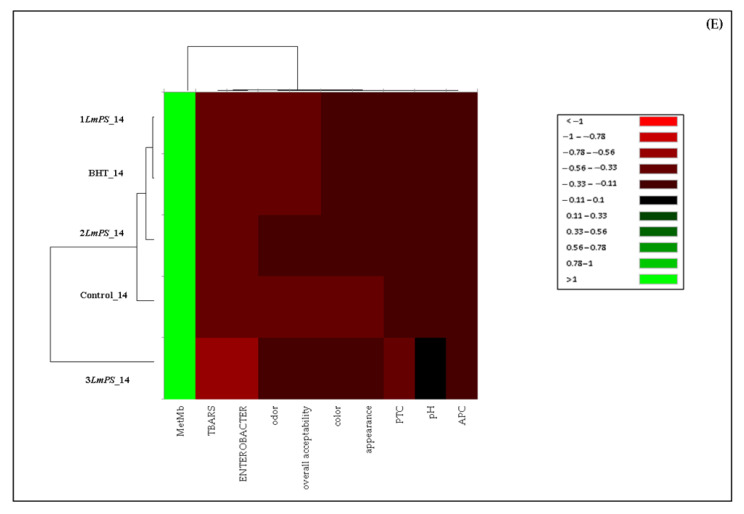
Two-way hierarchical cluster analysis (HCA) of physicochemical, microbial, and sensory characteristics of the control, BHT and different treated samples at each of the storage times: (**A**) day 0; (**B**) day 3; (**C**) day 7; (**D**) day 10 and (**E**) day 14.

Figure 4A shows three different clusters with a significant similarity between control and BHT samples. Thus, 1*Lm*PS*_0*, 2*Lm*PS*_0* and 3*Lm*PS*_0* showed dissimilarity in their composition. On days 3 and 7 of storage (Figure 4B,C), the dendrograms evidenced four groups with a high similarity between 1*Lm*PS (0.15% of *Lm*PS) and BHT samples. The 2*Lm*PS, control and 3*Lm*PS samples showed a different variation of the parameters depending on the refrigerated storage. Furthermore, the relationship among microbial contamination, lipid oxidation, protein oxidation, and sensory attributes was also highlighted by these two figures. Our results corroborate those of Kallel et al. [46], who stated that metMb (MbFe^3+^) can be reduced to native Mb (Fe^2+^) by *Lactobacillus* and *Staphylococcus*, and inhibits unsaturated fatty acid oxidation. Furthermore, an addictive relationship (metMb-sensory qualities) was shown on days 3 and 7 of storage. Nevertheless, on day 10 (Figure 4D) the relation was more marked between TBARS-*Enterobacteriaceae*, APC-PTC, and sensory qualities. Such data resulted in lipid oxidation, browning reactions caused by non-enzymatic processes, and myoglobin oxidation, which are considered the main factors influencing meat colour [55]. Ben Akacha et al. [6] related sensory properties to microbiological investigations. The high levels of microorganisms and lipid oxidation caused unsatisfactory sensory properties of the untreated meat. On the other hand, the samples treated with essential oil showed significantly satisfactory scores at the odour level due to the astringent properties [13]. Therefore, chemometric tools can be used to estimate the quality of meat products and their aging based on their colour properties and oxidative stability.

## 4. Conclusions

Natural food preservatives such as *Lm*PS are a promising alternative for extending the shelf life of chilled raw ground beef by minimizing bacterial growth rates. The present study showed that *Lm*PS (0.6%), an extract of *L. maritima*, inhibited lipid oxidation and bacterial growth during 14 days of storage at 4 °C in minced beef meat. *Lm*PS proved to be a potent food additive as stability of TBARS and metmyoglobin as well as pH was observed. It can also be considered a functional ingredient for improving shelf life and microbiological safety during refrigerated storage. Furthermore, *Lm*PS added to the meat samples at the concentration of 0.3% or above extended the sensorial shelf life of the meat stored in refrigerated conditions in comparison with BHT. PCA and HCA values suggest that lipid/protein oxidation parameters effectively correlated with the evolution of the microbial growth. All considered, *Lm*PS appears to be a promising candidate as an emulsifier and emulsion stabilizer as well as an antioxidant in meat products.

## Figures and Tables

**Table 1:** Experimental conditions for sample preparation.

	Acronym	Experimental Conditions
Lot 1	Control	untreated control
Lot 2	BHT	supplemented with 0.01% BHT
Lot 3	[1*Lm*PS]	supplemented with *Lm*PS at 0.15% (*v/w*)
Lot 4	[2*Lm*PS]	supplemented with *Lm*PS at 0.3% (*v/w*)
Lot 5	[3*Lm*PS]	supplemented with *Lm*PS at 0.6% (*v/w*)

All samples were stored at 4 °C for 14 days. Quality parameters were checked at days 0, 3, 7, 10 and 14.

**Table 2:** Antibacterial activity of *Lm*PS expressed as inhibition zones.

	Bacteria Strains	*Lm*PS	1/2	STR
G^+^	*Staphylococcus aureus* ATCC 25,923	21.5 ± 0.5	16.5 ± 0.5	13.5 ± 0.3
	*Listeria monocytogenes* ATCC 1911	31.5 ± 0.1	27.5 ± 0.5	27.0 ± 1.1
G^−^	*Escherichia coli* ATCC 25,922	29.5 ± 0.3	26.0 ± 1.2	27.5 ± 0.3
	*Salmonella enterica* ATCC 43,972	31.0 ± 0.3	27.0 ± 0.3	26.2 ± 0.8

*Lm*PS*: Lobularia maritima* polysaccharides (50 mg/well); 1/2: 25 mg/well *Lm*PS; streptomycin (STR) concentration: 20 μg/well. Values are mean ± S.E.M (*n* = 3) of triplicate experiments.

**Table 3:** Minimum Inhibitory Concentrations (MIC) and Minimum Bactericidal Concentrations (MBC) of *Lm*PS.

	Bacterial Strain	MIC (µg/mL)	MBC (µg/mL)	MBC/MIC	Antibacterial Activity
G^+^	*Staphylococcus aureus* ATCC 25,923	120 ± 0.01	150 ± 0.8	1	Bacteriostatic
	*Listeria monocytogenes* ATCC 1911	150 ± 0.04	400 ± 0.05	2	Bacteriostatic
G^−^	*Escherichia coli ATCC* 25,922	170 ± 0.05	400 ± 0.02	2	Bacteriostatic
	*Salmonella enterica* ATCC 43,972	150 ± 0.04	200 ± 0.01	1	Bacteriostatic

Values are mean ± S.E.M (*n* = 3).

**Table 4:** *Lm*PS effect on the microbial load of aerobic plate count (APC), psychrotrophic count (PTC), and *Enterobacteriaceae* count of crude minced meat beef stored at 4 °C.

Days of Storage at 4 °C					
	0	3	7	10	14
**APC**					
Control	2.0 ± 0.03 ^aA^	3.5 ± 0.23 ^dB^	4.9 ± 0.07 ^eC^	6.6 ± 0.33 ^dD^	8.0 ± 0.08 ^dE^
BHT	2.1 ± 0.11 ^aA^	3.2 ± 0.03 ^cA^	4.7 ± 0.49 ^dB^	6.1 ± 0.29 ^dC^	6.9 ± 0.07 ^cD^
1*Lm*PS	2.1 ± 0.01 ^aA^	3.2 ± 0.11 ^cB^	4.4 ± 0.35 ^cB^	6.2 ± 0.42 ^cC^	6.6 ± 0.38 ^cD^
2*Lm*PS	2.1 ± 0.04 ^aA^	3.2 ± 0.08 ^bB^	4.0 ± 0.31 ^bC^	5.1 ± 0.31 ^bC^	5.9 ± 0.28 ^bC^
3*Lm*PS	2.0 ± 0.04 ^aA^	2.8 ± 0.53 ^aB^	3.5 ± 0.07 ^aB^	4.1 ± 0.07 ^aC^	4.7 ± 0.42 ^aD^
**PTC**					
Control	2.0 ± 0.02 ^aA^	2.9 ± 0.6 ^dB^	3.8 ± 0.67 ^cC^	5.2 ± 1.40 ^cD^	6.4 ± 0.24 ^cE^
BHT	2.0 ± 0.23 ^aA^	2.7 ± 0.63 ^cB^	3.3 ± 0.14 ^cC^	4.7 ± 0.67 ^dCD^	6.0 ± 0.44 ^dD^
1*Lm*PS	2.0 ± 0.01 ^aA^	2.1 ± 0.02 ^bB^	3.2 ± 0.59 ^bBC^	4.7 ± 0.51 ^dC^	5.3 ± 0.55 ^bD^
2*Lm*PS	2.0 ± 0.54 ^aA^	2.1 ± 0.16 ^aA^	3.0 ± 0.25 ^aB^	3.7 ± 0.18 ^bC^	4.5 ± 0.21 ^aD^
3*Lm*PS	2.0 ± 0.05 ^aA^	2.0 ± 0.2 ^aAB^	2.6 ± 0.29 ^abB^	3.2 ± 0.25 ^aC^	4.1 ± 0.02 ^aC^
***Enterobacteriaceae* count**					
Control	<1 ^aA^	2.3 ± 0.02 ^bB^	2.6 ± 0.05 ^eB^	3.1 ± 0.66 ^cC^	3.5 ± 0.17 ^cC^
BHT	<1 ^aA^	2.1 ± 0.06 ^bB^	2.0 ± 0.14 ^dB^	2.5 ± 0.15 ^bC^	2.7 ± 0.15 ^bC^
1*Lm*PS	<1 ^aA^	2.0 ± 0.04 ^bB^	1.8 ± 0.18 ^cB^	2.2 ± 0.02 ^bC^	2.4 ± 0.27 ^aC^
2*Lm*PS	<1 ^aA^	1.9 ± 0.19 ^bB^	1.6 ± 0.14 ^bAB^	2.1 ± 0.05 ^aB^	2.3 ± 0.05 ^aB^
3*Lm*PS	<1 ^aA^	<1.0 ^aA^	1.2 ± 0.14 ^aA^	1.7 ± 0.14 ^aAB^	1.8 ± 0.29 ^aB^

Mean ± S.E.M (*n* = 3). Values with a different letter (a–e) of the same storage day are significantly different (*p* < 0.05). Values with a different letter (A–E) of the same concentration are significantly different.

## Data Availability

The datasets generated for this study are available on request to the corresponding author.
